# Grappling With the Inclusion of Patients and the Public in Consensus Building: A Commentary on Inclusion, Safety, and Accessibility: Comment on "Evaluating Public Participation in a Deliberative Dialogue: A Single Case Study"

**DOI:** 10.34172/ijhpm.2024.7715

**Published:** 2024-04-08

**Authors:** Davina Banner, Katrina Plamondon, Nelly D. Oelke

**Affiliations:** ^1^School of Nursing, University of Northern British Columbia, Vancouver, BC, Canada.; ^2^School of Nursing, University of British Columbia, Vancouver, BC, Canada.

**Keywords:** Deliberative Dialogue, Consensus Methods, Patient-Oriented Research, Inclusion, Equity

## Abstract

Deliberative dialogue (DD) may be relatively new in health research but has a rich history in fostering public engagement in political issues. Dialogic approaches are future-facing, comprising structured discussions and consensus building activities geared to the collective identification of actionable and contextualized solutions. Relying heavily on a need for co-production and shared leadership, these approaches seek to garner meaningful collaborations between researchers and knowledge users, such as healthcare providers, decision-makers, patients, and the public. In this commentary, we explore some of the challenges, successes, and opportunities arising from public engagement in DD, drawing also upon insights gleaned from our own research, along with the case study presented by Scurr and colleagues. Specifically, we seek to expand discussions related to inclusion, power, and accessibility in DD, highlight the need for scholarship that addresses the epistemic, methodological, and practical aspects of patient and public engagement within dialogic methods, and identify promising practices.

 Recently, focus on collaborative methods and meaningful collaboration between researchers and knowledge users to address pressing health and social issues has increased.^1^ Deliberative dialogue (DD) is one such approach that explicitly values the engagement of those with diverse perspectives and experiences.^1^

 DD may be relatively new in health research, but has a rich history in public engagement.^2,3^ Dialogic approaches are future-facing and comprise structured activities geared to the collective identification of actionable and contextualized solutions.^4^ DD is underpinned by four characteristics: (1) consultation with knowledge users affected by the issue; (2) fair and purposeful representation of key knowledge users; (3) high quality summary of relevant evidence; and (4) skillful facilitation.^5^ These methods may be employed to address significant issues for public or specific populations where evidence alone is insufficient to address an issue, there may be imbalances in knowledge, or there is a need for resolution.^5,6^

 Scurr and colleagues^7^ provide a detailed evaluation of public participation in a DD to improve the social environment and decrease social isolation in a rent-geared-to-income housing complex. Using a case study design, comprising a survey and focus groups, they examined how integration of community tenants impacted the DD process, provided a deep dive into the DD process, and complexity of engagement. They reinforce this complexity, noting that DDs are not “one-size-fits-all” (p. 2)^7^ and highlight the challenge of evaluating processes and outcomes.

 Robust evaluations contribute much needed insight into this developing field. By illuminating key methodological considerations and nuanced practices, these show potential to advance the science of consensus methods,^5^ along with other modes of patient and public engagement, including patient-oriented research (POR). Here, we explore challenges, successes, and opportunities arising from DD, drawing also upon insights gleaned from our own research exploring consensus methods in POR. Specifically, we expand discussions related to inclusion, power, and accessibility, highlighting the need for scholarship that addresses the epistemic, methodological, and practical aspects of patient and public engagement within DD.

## Fostering Meaningful Inclusion and Engagement: Considerations of Perspective and Power

 DD requires engagement of individuals with diverse perspectives and experiences, ideally, by *anyone* affected by an issue.^5^ These aspirations do not mean that the method *de facto *leads to authentic engagement or representation. Issues of representation are subject to societal power dynamics and assumptions, which are deeply rooted in structures of exclusion that elevate and privilege already advantaged voices.^8,9^ DD is, in itself, a risk-taking approach: if done well, it brings people into places where they solve problems together. This method convenes people around a shared issue, builds collective understanding, and works to co-create actionable solutions. While dialogic methods offer transformative potential, intentional strategies are needed to counter dominant positionalities and voices.^10^

 Scurr and colleagues provided rich descriptions of engagement processes, including the convening of a steering committee to guide study decision-making and facilitate participant recruitment and engagement.^7^ They note that those participants whose input would be most valuable would likely be most difficult to recruit: a common, yet critical, challenge. Without purposeful attention to inclusion, teams risk accepting or reproducing systems of exclusion.

 Equity-informed research approaches promote participation by meeting people *where they are, *in ways that honour their contributions, and through inclusive, safe, and respectful environments. However, the evaluation outcomes here uncover inclusion challenges, including venue acceptability and comfort and safety of the engagement process. In our own research, inclusion remained a central consideration. We sought to understand how diverse patient and public communities can be best engaged in DD, explicitly seeking to identify meaningful linkages between equity-informed methods and practices. We suggest there are specific considerations and practices that can more authentically promote inclusion. Some of these fall under what is ‘soft’ or even, “*merely administrative” *or “*logistical.*” One of our research participants wisely pointed to the ways in which the “*research lens”* is often required to legitimize knowledge and practice, requiring it to be filtered through the academic process before being considered valuable.

 In a community that, as Scurr and colleagues reported, already experienced harm from research, there were very real, ethically critical considerations that were navigated. Intentions for meaningful engagement were clearly infused, yet elements arose where the full potential for DD may have been missed. These can be hard to catch in the moment, but often become more visible in retrospect, supporting the benefits and potential learnings that can be drawn from rich evaluations.

 Dialogic processes are not neutral and grounded in structures and systems of power.^10,11^ Teams must craft balance among the diverse perspectives as a means of moving to a space of action. For example, a particularly large proportion of one stakeholder/power holding group may imbalance procedures, while populations that face barriers to engagement may require extended periods of trust building and engagement. In either case, a failure to support inclusion will adversely impact the outcomes. Issues related to balance emerged in the study by Scurr, where imbalances arose between the goals of tenants and those of the researchers and other knowledge users. In the case provided, the authors noted that the experiences and narratives of tenants “*strayed from the agenda and issue brief,” *but space was created as a* “platform to air their frustrations*” (p. 8).^7^ Providing meaningful opportunities for participants to share perspectives early in the process, such as undertaking qualitative interviews or focus groups, can be valuable. This can foster more contextualized evidence and allow for greater focus on action and resolution.

 Inclusion practices require deep attentiveness to time and resources that enable *authentic* and *meaningful* engagement. Issues of power are important when considering the engagement of multi-stakeholder groups, in this case, community tenants, those who provide housing services, public health providers, and researchers. Within the case, we are exposed to the complexities of power and voice. Of note, some professional participants were operating within positions of power that could potentially result in negative impacts for community tenants. In the article, the authors highlight the extensive planning underpinning this process, including the explicit and intentional process of confronting power relationships and negotiating mitigation strategies. However, in Table six, we see evidence of concerns related to positionality and power, as one tenant commented “*I should have a bag over my head and…been more anonymous …[ the DD] has brought me to the forefront and to the attention of management*”(p. 9).^7^ Likewise, some professional stakeholders felt the need to hold back to create space for community tenants to share experiences.

 Teams must navigate power through an ethical lens, carefully weighing the risks and benefits of research and explicitly considering who bears the burden of those risks. Harmful interactions or negative outcomes can foster distrust, relational harm, and/or direct conflict, in ways that are more likely to resonate through tenants’ lives (whose daily living conditions are tied up in the topic and *location* of dialogue) than in researchers’ (who leave the *place* of dialogue and enter into another social world). Thus, even when the intention is more authentic engagement, there can be inequitable and potentially harmful consequences to any form of research co-production.^10,11^ Careful attention to relationships and relational practices is needed.

 Trust, reciprocity, mutuality, and creating spaces of welcome are the essential practices of inclusion. Emphasis must be placed on providing the time and platform needed by the people *most affected by decisions of other* to voice their experiences in ways that work for them. Timelines, funding, and venues all become central considerations that in turn may constrain what is feasible. Teams must navigate issues of power with sensitivity, working to co-create appropriate mitigation strategies. This may include working collectively to identify and name power imbalances, co-develop strategies that foster inclusion and safety (eg, group values or agreements), or at times consider the integration of those in boundary roles who may be more protected against adverse outcomes. In our own research, we learned that even the act of introductions plays a role in cultivating an inclusive place for dialogue. Inclusive DD facilitation techniques invite more personal connections among participants, without requiring only particular people, usually the “patient partner” or “community participant,” to foreground vulnerabilities.

## Equity-Informed Knowledge Mobilization – A Call for Accessibility

 Dialogues are centrally organized around knowledge exchange and translation, however, knowledge is not neutral. Plamondon and colleagues remind us that “*Research is never benign and always politica*l” (p. 37).^12^ How knowledge is created, owned, valued, and shared is inherently political and typically grounded in western epistemic and ontological perspectives. Here, we draw attention to opportunities for effective knowledge mobilization, including the use of tailored DD materials that promote accessibility and inclusion.^13^

 Evidence briefs are a vital DD tool, used to summarize key issues and support meaningful deliberation.^2,6^ Traditionally textual documents, these are grounded in scientific evidence,^14,15^ but may include expert opinion or contextualized insights.^6^ Perspectives related to knowledge form a critical foundation in how evidence briefs are used and valued. In the context of DDs, particularly those with a broader inclusion lens, teams are challenged to consider the contestable nature of evidence, being required to reflect upon how distinct knowledge is created or privileged, and what perspectives may be absent or silenced. In the article, the authors detail the creation of a 35 page evidence brief, including the use of rapid synthesis methods and integration of the core working group perspectives and community health profile. A recognized risk of this approach is that the focus of the DD may be prematurely determined and may not reflect the broader topics of interest or concern.

 Concerns regarding the legitimacy, privileging, and sharing of knowledge are faced widely within health and social research. Teams commonly have restricted resources and time, requiring a focused or narrowed engagement process. This may limit the potential reach of the work or perpetuate over-representation of dominant voices.^14^ Like Scurr and colleagues, providing detailed descriptions of engagement, along with potential limitations in inclusion and scope, can highlight areas for future engagement. Researchers are inherently bound by their own assumptions and positionality, necessitating the ongoing need for critical reflexive practices. When researchers engage with an absence of relational accountability,^15^ the full potential of engagement methods may be missed. Accessible and evidence-informed knowledge mobilization strategies are needed to support inclusion within DD.

 In this case study, we learn of the varied accommodations to optimize inclusion, including the co-creation of a plain language evidence brief and orientation for community tenants. Like Scurr and colleagues, we too sought to generate evidence products, including evidence briefs, that were accessible across diverse audiences. Where possible, limiting their length and including plain language resources, including glossaries, infographics, and summaries. During some of our early DDs, we noted that some participants attended having not reviewed, or only briefly skimmed, preparatory resources. To maximize engagement, we offered patient/community participants a brief orientation session, along with teaser videos that detailed the DD approach and key highlights from the evidence brief. Overall, we found the inclusion of a variety of knowledge mobilization strategies served to increase preparation and support inclusion.

## Moving Forward: Gaps and Opportunities

 Dialogic methods can generate contextualized and evidence-informed strategies that tackle complex health and social issues. By attending to issues of inclusion, power, and accessibility, teams have the potential to disrupt dominant discourses, offering a way to counter, confront, and raise awareness of inequities. Continued focus upon the philosophical, methodological, and practical aspects of such approaches is warranted and provides valuable pathways to more inclusive and equitable health research.

 Scurr and colleagues evaluated a DD process aimed at improving social outcomes for community tenants.^7^ This work highlights the complex theoretical and practical tensions that must be addressed when engaging with populations that face barriers or experience marginalization. The authors offer thoughtful insights, illuminating several successes, challenges, and opportunities. This is a processual element that is often missing from scientific accounts of DD. In this commentary, we have offered some additional considerations and insights relevant to inclusion, power, and accessibility, elements that most teams grapple with. Ongoing efforts to evaluate how inclusionary practices foster transformative impact is needed, along with targeted DD reporting guidelines and evaluation tools. Addressing this will advance dialogic methods and generate useful knowledge across broad research paradigms.

## Ethical issues

 Not applicable.

## Competing interests

 Authors declare that they have no competing interests.

## Funding

 In this commentary, we draw upon insights from our Canadian Institutes of Health Research/BC ‎SUPPORT Unit funded study exploring consensus methods in POR.‎

## References

[1] Gagliardi AR, Dobrow MJ (2016). Identifying the conditions needed for integrated knowledge translation (IKT) in health care organizations: qualitative interviews with researchers and research users. BMC Health Serv Res.

[2] Lavis JN, Boyko JA, Oxman AD, Lewin S, Fretheim A (2009). SUPPORT tools for evidence-informed health Policymaking (STP) 14: organising and using policy dialogues to support evidence-informed policymaking. Health Res Policy Syst.

[3] Burgess MM (2014). From ‘trust us’ to participatory governance: deliberative publics and science policy. Public Underst Sci.

[4] Boyko JA, Lavis JN, Dobbins M (2014). Deliberative dialogues as a strategy for system-level knowledge translation and exchange. Healthc Policy.

[5] Lomas J (1986). The consensus process and evidence dissemination. CMAJ.

[6] Morain SR, Whicher DM, Kass NE, Faden RR (2017). Deliberative engagement methods for patient-centered outcomes research. Patient.

[7] Scurr T, Ganann R, Sibbald SL, Valaitis R, Kothari A (2022). Evaluating public participation in a deliberative dialogue: a single case study. Int J Health Policy Manag.

[8] Banner D, Bains M, Carroll S (2019). Patient and public engagement in integrated knowledge translation research: are we there yet?. Res InvolvEngagem.

[9] Pauly B, Revai T, Marcellus L, Martin W, Easton K, MacDonald M (2021). “The health equity curse”: ethical tensions in promoting health equity. BMC Public Health.

[10] Rycroft-Malone J, Wilkinson J, Burton CR (2013). Collaborative action around implementation in Collaborations for Leadership in Applied Health Research and Care: towards a programme theory. J Health Serv Res Policy.

[11] Langley J, Wolstenholme D, Cooke J (2018). ‘Collective making’ as knowledge mobilisation: the contribution of participatory design in the co-creation of knowledge in healthcare. BMC Health Serv Res.

[12] Plamondon K, Banner D, Cary MA (2023). Relational practices for meaningful inclusion in health research: Results of a deliberative dialogue study. Health Expect.

[13] Chambers D, Wilson PM, Thompson CA, Hanbury A, Farley K, Light K (2011). Maximizing the impact of systematic reviews in health care decision making: a systematic scoping review of knowledge-translation resources. Milbank Q.

[14] Bohm D, Senge P, Nichol L. On Dialogue. Routledge; 2004.

[15] Doane GH, Varcoe C (2007). Relational practice and nursing obligations. ANS Adv Nurs Sci.

